# Malignant Disease of the Tonsil

**Published:** 1876-09

**Authors:** J. F. Miner


ART. II.—*Malignant Disease of the Tonsil; Removal by Galvano-Cautery.* By Prof. J. F. MINER, M. D. Reported by E. N. BRUSH, M. D.

On the second of July last, Mr. B. of Nunda, N. Y., came to consult Dr. Miner in reference to an enlargement of his right tonsil, which seriously interfered with both respiration and deglutition, and was also producing considerable annoyance from the amount of pain which it produced. The patient, a man of about sixty-three years, presented an anxious, cachectic appearance, and though of an apparently sturdy constitution, he now looked ill and worn out. An examination of the throat revealed a tumor springing from the seat of the right tonsil and almost filling the entire throat. It was nodulated in appearance, soft, and bled on slight manipulation. The discharge from the surface was not very profuse, but had a disagreeable odor. The glands in the neck on the corresponding side, were enlarged. The tumor in the throat had been of somewhat rapid growth, the exact duration of which was not, however, at the time, noted. No family history of cancer was elicted.

The diagnosis made was encephaloid disease of the tonsil, and after carefully weighing the dangers, of the operation, its removal was advised, not with the idea of a permanent cure, but for the purpose of affording temporary relief, which was all that Mr. B. expected. The following day, July 3d, was fixed for the operation.

*Operation.* Dr. W. W. Miner, having induced anæsthesia by ether, the operation was commenced, with the assistance of Dr. W. W. Miner, the writer, Messrs. Hopkins and Parker, private students of Prof. Miner's. An attempt was first made to pass the wire loop of the galvano-cautery apparatus about the base of the tumor, but owing to the shape of the growth and its situation, this was found impossible. The platinum knife was next heated red hot, and the larger portion of the tumor shaved off with it. It was found impossible, without injury to the surrounding tissues, to remove the entire tumor in this manner, and resource was had to a pair of long curved scissors, by which all remaining fragments of the tumor were removed. The hemorrhage from the cut surface, which was quite free, was easily controlled by the dome cauteriser heated by the battery. The surface left, after the removal of the tumor and application of the cantery, had a smooth, even feeling, and was evidently in good condition for whatever reparative attempt nature might make. The enlarged glands in the neck were removed by the knife.

On the 15th of September, Mr. B. again presented himself for examination and advice. The tumor in the throat had not again made its appearance, but the site of the original growth had an unhealthy look. In the neck the glands had enlarged, and at the site of those removed a tumor had attained the size of an English walnut. No operative procedure was advised, the object of the original operation having been attained, viz :—removal of the obstruction to deglutition and respiration.

Malignant disease of the tonsil is not of very frequent occurrence. There is nothing, however, in this case, which warrants a report of its details, except the facility with which the tonsil was removed. The risks attending an operation of this character, and upon which some surgeons have laid considerable stress, have, heretofore, been in the danger of uncontrollable hemorrhage, but surgeons now have at their command an apparatus by which the actual cautery can be applied with ease, precision, and at a moments notice, and by the employment of the galvano-cautery the danger surrounding this operation is in a great measure removed. The instrument used in this instance was what is known as the Byrne battery. It seems to possess all the advantages of the larger batteries, and one other which they do not, namely, portability, ocupying as it does less space than an ordinary amputating case.

